# The Control of Lay Women in Hospitals

**Published:** 1916-08-05

**Authors:** 


*To the Editor of* The Hospital.

### The Control of Lay Women in Hospitals.

*To the Editor of* The Hospital.

Sir,—I am interested in "Matron's" letter not because I agree with her suggestion that there is any need for the control she suggests, but because it affords an opportunity for opening a question in respect of these women lay workers which I believe is difficult to answer. As to control, the women are in distinct departments—the pharmacist's, the secretary's, the out-patient superintendent's, and the like, and there appears to be little room for control that the head of the department cannot exercise. My trouble is the question of dress. A hospital is a sombre and grave place, the dress of most of the workers is severe in character, the patients are very seldom dressed in bright colours. But when it happens that these lay workers, clerks, dispensers, etc., flit about like butterflies in fashionable attire, with the V-shaped opening to the blouse, with silk stockings and short skirts, with fancy-top boots (such as munition workers like), with rings on their fingers and chains round their necks, a harsh note is struck. No doubt the same difficulty occurs in banks, etc., but there the atmosphere is different. Does the solution lie in overalls? Even then the lay woman worker does not cover up her silk stockings, her fancy boots, and her jewellery. Does it answer if you instruct them, when engaging their services, to dress quietly? I have not been helped in this way, because opinions differ very widely as to what is quiet. Please can somebody tell me what to do?—Yours faithfully,

BASHFUL SECRETARY.

